# Exploring the Cutting Edge: A Recap of EULAR & ACR 2023 Highlights for Spondyloarthritis, including Psoriatic Arthritis

**DOI:** 10.31138/mjr.040524.aeh

**Published:** 2024-12-31

**Authors:** Nafsika Gerolymatou, Paraskevi V. Voulgari

**Affiliations:** Department of Rheumatology, School of Health Sciences, Faculty of Medicine, University of Ioannina, Ioannina, Greece

**Keywords:** SpA, PsA, ACR, EULAR

## Abstract

As the field of rheumatology continues to advance, the European Alliance of Associations for Rheumatology (EULAR) and the American College of Rheumatology (ACR) annual meetings stand as pivotal events, showcasing the latest research, treatments, and insights into various rheumatic diseases. Among these, Inflammatory Spondyloarthritis presents a unique challenge, characterised by its elusive diagnostic markers and diverse clinical manifestations. In this article, we delve into the highlights and findings from EULAR 2023 and ACR 2023, shedding light on the latest advancements in understanding and managing these complex rheumatic conditions.

## INTRODUCTION

This review systematically compiled and analysed the latest research presented at the European League Against Rheumatism (EULAR) 2023 and the American College of Rheumatology (ACR) 2023 conferences. Initially, a comprehensive database search was conducted, focusing on abstracts and presentations from both conferences. Keywords related to key themes of the conferences, such as “psoriatic arthritis”, “axial spondyloarthritis”, “spondyloarthritis”, and “seronegative inflammatory arthritis ” were used to filter relevant studies.

Within the abundance of abstracts and publications presented at EULAR 2023 and ACR 2023, a notable trend emerged: a significant emphasis on psoriatic arthritis (PsA) within the spectrum of seronegative inflammatory arthritis. This focus reflects the growing recognition of PsA as a distinct entity with unique clinical features and therapeutic considerations.

## BASIC SCIENCE EULAR 2023

In order to study gene expression and gene signalling pathways involved in B cell activation in ankylosing spondyloarthritis (AS), investigators from the Netherlands analysed the transcriptomic profile of isolated peripheral B cells from AS patients, healthy donors (HD), and patients with primary Sjögren syndrome (pSS), a typical B-cell-associated autoimmune disease. RNA was isolated from CD19+ B cells isolated from peripheral blood mononuclear cells of 8 AS patients, 8 sex and age-matched HDs, and 8 age-matched pSS patients. Analysis of differential gene expression between AS patients and HDs revealed upregulation of 8 genes, including MAPK14, KMT2A and PKM, in B cells from AS patients, and downregulation of two genes, DDIT4 and ATG5.

In AS patients, the expression levels of genes related to BCR signalling and Fc receptors and phagocytosis pathways were significantly higher compared to healthy donors. Numerous differentially expressed genes involved in B cell signal transduction pathways were identified, alongside an observed increase in genes associated with B cell activation. These findings imply that B cells actively participate in the disease process of AS.^1^

A study from Hungary examined the effects of kynurenic acid (KYNA) analog in AS and PsA. The investigators examined the effects of a newly synthesised KYNA analog on blood samples from rheumatoid arthritis patients, focusing on TNF-α, S100A8/9, S100A12 alarmins, α-defensin, and IL-17 production. They correlated these effects with the expression of TNF-stimulated gene-6 protein (TSG-6) in AS and PsA patients. Peripheral blood mononuclear cells were isolated from blood samples of 38 PsA patients, 54 AS patients, and healthy donors. TSG-6 expression may help suppress inflammatory cytokines, such as TNF, calprotectin, and α-defensin, in spondyloarthropathies. The production of TSG-6 by the KYNA analog could be a mechanism behind its anti-inflammatory effect, making it a potential target for therapeutic applications in AS and PsA.^2^

## BASIC SCIENCE ACR 2023

Raimondo M et al. utilised a pre-clinical model of PsA and psoriasis (PsO) to elucidate the differences in the pathophysiology between these two diseases. Approximately 30% of PsO patients develop PsA over time, suggesting a disease-mediated skin-joint crosstalk. However, it remains unclear why the inflammatory process in some PsO patients is confined to the skin, while in others it spreads to the joints. To address this issue, the researchers employed an innovative approach using an IL-23 overexpression (IL-23OE) mouse model of PsO. This model, conducted across various genetic backgrounds of KAEDE-transgenic mice, facilitated the assessment of cell migration from inflamed skin to the joints, providing crucial insights into the intricate interplay between cutaneous and articular manifestations of psoriatic disease.

Their findings revealed that psoriatic skin lesions were induced by IL-23 overexpression regardless of the mouse strain, while the onset of joint inflammation was influenced by the genetic background of the mice. Single-cell RNA-sequencing (scRNAseq) indicated CD2+ MCHII+ monocytes as the predominant cell type evading from the inflamed skin and entering the synovial tissue, with no frequency differences between PsO and PsA mice. Furthermore, the interaction of these monocytes with stromal-resident cells led to the emergence of two distinct phenotypes, characterised by pro-inflammatory signatures in mice developing PsA.^3^

Previous research has shed light on the role of the microbiome in PsA, revealing a notable decrease in gut microbiome diversity among PsA patients. This decline mirrors the dysbiosis observed in inflammatory bowel disease, hinting at potential shared mechanisms between these conditions. Furthermore, the gut dysbiosis in PsA patients correlates with reduced levels of protective fatty acids. These fatty acids are crucial for promoting the proliferation of regulatory T cells in the gut, a process that effectively suppresses pro-inflammatory Th17 cells, thus serving as a protective mechanism against gut inflammation.^4^ In a recent study by Paine et al., new insights emerged regarding serum metabolites in PsO patients who subsequently developed Psoriatic Arthritis. Notably, the levels of butyrate, a short-chain fatty acid (SCFA), were found to be significantly decreased even before the onset of arthritis. This early reduction in butyrate levels suggests a potential metabolic signature associated with the progression from psoriasis to psoriatic arthritis, underscoring the importance of metabolic dysregulation in the pathogenesis of these conditions.^5^

## CLINICAL RESEARCH EULAR 2023

### Spondyloarthritis – Clinical aspects

A cross-sectional, multicentre study was conducted in five university hospitals in France to evaluate the prevalence of irritable bowel syndrome (IBS) in patients with axial spondyloarthritis (axSpA). Both patients and rheumatologists completed questionnaires. The study included a total of 500 patients (47% women, mean age 49.5±13.8 years, mean disease duration 14.7±11 years, and mean BASDAI 3.6±2.1). Among the patients, the vast majority (86%) received TNF inhibitors, 13% IL-17 inhibitors, 1% IL-12/IL-23 inhibitors, and 25% NSAIDs. IBS was present in 124 patients (25%) and was found to be associated with female gender, fibromyalgia, anxiety, and depressive disorder. Patients with IBS appear to have a more challenging disease course, characterised by higher activity, worse functional scores, and multiple lines of treatment.^6^

The International Map of Axial Spondyloarthritis (IMAS) study, a cross-sectional online survey (2017–2022) of 5557 unselected axSpA patients from 27 countries, evaluated gender differences in axSpA patients. Females with axSpA exhibited better overall health behaviours. However, female patients were less likely to be HLA-B27 positive and experienced a longer diagnostic delay. Although being subscribed medication more frequently, females with AxSpa had higher disease activity, worse functional outcomes, and poorer mental health.^7^

A post hoc analysis of week 52 results from the phase 3 studies BE MOBILE 1 (non-radiographic axSpA [nr-ax-SpA]) and BE MOBILE 2 (radiographic axSpA [r-axSpA]) included patients irrespective of their initial treatment arm (placebo then BKZ from week 16 or continuous BKZ 160 mg Q4W). Most patients completed week 52 (nr-axSpA: 220/254, 86.6%; r-axSpA: 298/332, 89.8%). Patients who met more strict clinical response criteria and had lower levels of disease activity at the end of the study periods, also exhibited a bigger amelioration in outcomes such as physical function, sleep quality, and HRQoL.^8^

A retrospective, population-based, cross-sectional study determined the prevalence of heart valve disorders in AS patients. The study included 4082 AS patients and 20.397 age- and sex- matched controls. AS patients had a significantly higher prevalence of cardiovascular risk factors (p<0.001) and a higher prevalence of valvular heart disease. In the multivariate logistic regression analysis, AS was independently associated with aortic stenosis (OR 2.25, 95% CI 1.57–3.23, p<0.001), aortic insufficiency (OR 2.44, 95%CI 1.5–3.94, p<0.001) and mitral insufficiency (OR: 1.75 95% CI 1.17–2.61, p<0.001). The heart valves are considered enthesis-like structures, subject to increased biomechanical stress and inflammation in patients with AS, possibly associated with the increased risk of valvular heart diseases observed in this population.^15^

### Psoriatic arthritis – Treatment

Patients with PsA and inadequate response to TNF inhibitors (TNFi-IR) receiving guselkumab in the phase 3b COSMOS trial (189 patients) were included in the analysis to assess the association between early clinical response across various PsA domains and HRQoL at week 48. ACR 20 responses at week 4 and week 8 was positively associated with SF −36 physical component summary improvement from baseline to week 48 and at week 8 with Dermatology Life Quality index improvement from baseline to week 48. Therefore, early clinical response is relevant for HRQoL improvements over time, offering help in shared decision -making processes.^9^

A post hoc analysis of DISCOVER-2 which included patients with active PsA naïve to biologics/JAK inhibitors evaluated the long-term effect of guselkumab in maintaining or further improving fatigue between week 52 and week 100 using the Functional Assessment of Chronic Illness Therapy – Fatigue (FACIT-F) scale. Clinically significant improvements in fatigue were seen after 1 year on guselkumab treatment and were further augmented over two years, with nearly a third of treated patients presenting normative FACIT-F levels by that time.^10^

A post-hoc analysis of 25 RCTs, investigated the long-term incidence rates (IRs) of malignancies in adults with PsO, PsA, or axSpA who received at least one dose of ixekizumab (IXE) in clinical trials. The analysis included 6,892 patients with PsO, 1,401 patients with PsA, and 932 patients with axSpA. The incidence rates (IRs) of malignancies at 1-year intervals were maintained low (≤1.2/100 PY) and stable for up to 5 years in PsO patients and up to 3 years in PsA and axSpA patients.^11^

Similarly, another post hoc analysis examined the long-term safety outcomes related to major adverse cardiovascular events (MACE) in adult patients with PsO, PsA, and axSpA who received at least one dose of IXE over 5 years (PsO) or 3 years (PsA, axSpA). The incidence rate of MACE was estimated to be low and stable over the treatment periods examined (IR=0.5 for PsO patients, IR=0.5 for PsA patients, and IR=0.3 for axSpA patients).^13^

A post hoc analysis of DISCOVER-2 data evaluated the long-term efficacy (week 100) of guselkumab in achieving stringent disease control across domains recommended by the Group for Research and Assessment of Psoriasis and Psoriatic Arthritis (GRAPPA), considering various baseline characteristics. Minimal disease activity (MDA) response at week 100 was achieved across various baseline characteristics, and the proportion of responders demonstrated non-significant differences across patient subgroups or between guselkumab dosing regimens. Results were similar regarding achievement of other outcomes (ACR50, ACR70, investigator’s Global Assessment score of 0 [IGA 0], Psoriatic arthritis Disease activity score-low disease activity [PASDAS LD], enthesitis resolution, dactylitis resolution, FACIT-F response, and HAQ-DI response). These findings support the long-term efficacy of guselkumab across a wide range of PsA disease domains and diverse PsA populations.^12^

Treatment effects of IXE and adalimumab (ADA) at the individual digit level with nail and distal interphalangeal joint involvement in patients with PsA were studied in a post hoc analysis of SPIRIT-H2H study. 354 patients with a NAPSI total score >0 and DIP involvement in at least 1 digit simultaneously at baseline were treated with IXE (N=186) or ADA (N=168). Patients treated with IXE had less nail involvement, less DIP involvement and less tenderness compared to those treated with ADA at week 24.^14^

### Spondyloarthritis – Treatment

A systematic review of literature and meta-analysis on the impact of different biologic disease-modifying antirheumatic drugs (bDMARDs) on patient reported outcomes in axial spondyloarthritis (axSpA) included 25 studies with a total number of 8457 patients. bDMARDs, including anti-tumour necrosis factor and anti-inter-leukin-17, significantly improve the outcomes of pain, fatigue, and quality of life compared to placebo. No significant differences were detected between anti-TNF and anti-IL-17 treatments. Similar findings were observed between the radiographic and non-radiographic axSpA groups, except for the mental component of the SF36, which showed less improvement in the non-radiographic axSpA.^16^

A post hoc analysis of pooled data from placebo-controlled randomised, double-blind trials in patients with ankylosing spondylitis (AS) who received at least one dose of tofacitinib or placebo (placebo treated patients switched to tofacitinib after week 16), showed that tofacitinib was more efficacious versus placebo and across endpoints regardless baseline CRP (normal <5 mg/L, elevated ≥5 mg/L). Tofacitinib safety rates were comparable to placebo in the elevated CRP group of patients but tended to be higher for tofacitinib versus placebo on patients with normal values.^17^

Another study examined the safety profile of bimekizumab (BKZ) at week 16, including the results from four placebo-controlled phase −3 studies, BE MOBILE 1 and BE MOBILE 2 in axSpA as well as BE OPTIMAL and BE COMPLETE in psoriatic arthritis (PsA). Bimekizumab (BKZ) demonstrated an overall safety profile consistent with previous phase 2 trials in axSpA and PsA, with a low rate of discontinuation. As expected, a higher rate of oral candidiasis compared to placebo was observed.^18^

A meta-analysis was conducted in China to evaluate the efficacy and safety of iguratimod on active axSpA. Iguratimod is a newly developed small molecular anti-rheumatic drug, which can affect the prostaglandin E2, TNF-Ş and IL-17. Seven randomised controlled studies were eligible for the meta-analysis, including 208 patients in the iguratimod group and 186 cases in the control group. The improvement of back pain, morning stiffness, BASDAI, ASAS 20, 40, ESR and CRP in the Iguratimod treatment group were better than those in the control group. There was no significant difference in the reported adverse events between the two groups.^19^

A systematic review and meta-analysis examined the effectiveness of IL-23 inhibitors on axSpA and axial PsA. Nine studies were eligible for inclusion, 5 examining axSpA and 4 studies for axial PsA. The use of IL-23 inhibitors was not supported in axSpA based on the analysed outcome measures of interest. The results of the three ustekinumab trials demonstrated that the drug was not efficacious in treating axSpA. However, trials in axial PsA investigations showed an improvement in BASDAI, modified BASDAI, and ASDAS. This meta-analysis raises several questions regarding differences in pathogenesis of axSpA and axial PsA.^20^

Another study from Spain evaluated the low spinal radiographic progression after a mean of 15 years of follow-up in a cohort of patients with axSpA. A total of 77 patients (53 were male), naïve to bDMARD at baseline, were included in 2006–2007 in the multicentre Spanish registry REGISPONSER and were re-evaluated in 2021–2022 using spine (cervical and lumbar) radiographs. The mean and median progression estimated were 0.54 and 0.38 points in mSASSS (modified stoke ankylosing spondylitis spinal score) per year respectively. Interestingly, the rate of progression in this established axSpA population was lower that what has been reported in similar cohorts. Presence of low back pain was the only disease factor associated with spinal radiographic progression.^21^

### Clinical Research ACR 2023

In a study led by Eder et al., an investigation into the cellular immune profiles of patients with PsA revealed possible correlations with both imaging patterns and treatment outcomes. Employing mass flow cytometry to characterise immune cell populations in whole blood, the researchers identified three distinct immune cell clusters. These clusters were not only linked to patients' baseline characteristics but also demonstrated associations with clinical and sonographic responses to therapy. Specifically, CD4+ memory and Th1 cells were found to be correlated with more severe synovitis and enthesitis, as well as poorer responses to b/tsDMARDs. Conversely, γδT cells and CD8+ naïve cells were associated with a milder disease phenotype and enhanced treatment response, offering valuable insights into potential biomarkers for treatment stratification in psoriatic arthritis.^22^ The current guidelines for PsA emphasise the importance of early initiation DMARDs, ideally within a maximum delay of one year from symptom onset. However, despite these recommendations, diagnostic delays of 1 to 2 years are still prevalent among PsA patients. In an effort to explore the existence of a therapeutic “Window of Opportunity” akin to that established for Rheumatoid Arthritis (RA) patients (i.e., disease duration <12 weeks before treatment initiation), researchers from the Netherlands, led by Snoeck Henkemans et al., conducted a comprehensive investigation.^23^

Their study, involving 855 newly diagnosed PsA patients, categorised participants into three groups based on diagnostic delay: <12 weeks, 12–52 weeks, and >52 weeks. Interestingly, the group experiencing a delay of more than 52 weeks was significantly less likely to achieve Minimal Disease Activity (MDA) and Disease Activity in Psoriatic Arthritis (DAPSA) remission over a three-year follow-up period compared to both the early and late diagnosis groups. Surprisingly, radiographic progression did not demonstrate significant differences between the groups. Although all outcomes tended to be numerically superior in PsA patients diagnosed within 12 weeks compared to those diagnosed within 52 weeks, the study did not find statistically significant differences between these groups. This highlights the potential importance of timely diagnosis and treatment initiation in improving long-term outcomes for individuals with PsA.^23^

As extensively discussed in the literature, a significant number of patients with psoriatic arthritis (PsA) remain resistant to advanced therapeutic interventions and are classified as difficult-to-treat (D2T) PsA. Nevertheless, there is considerable variation in defining active disease and no consensus on the number of treatments a patient must fail before being classified as D2T PsA. Of note, the EULAR and GRAPPA societies have recently initiated two parallel projects aiming to establish a widely accepted definition of “difficult-to-treat” PsA.^31^

In alignment with current Rheumatoid Arthritis guidelines and the concept of “D2T RA” (Difficult-to-treat RA), a group of French researchers set out to define a similar subset within Psoriatic Arthritis (PsA) and Axial Spondyloarthritis (AxSpA) patients. Their objective was to characterise patients who required multiple treatment switches within a short timeframe due to inadequate response. Conducting two separate retrospective studies, one for PsA and the other for AxSpA patients, the researchers included individuals who had initiated biologic or targeted synthetic DMARDs (b/tsDMARDs) with a minimum two-year follow-up period.^24,25,30^

“D2T PsA” was defined as the failure of two or more b/tsDMARDs with distinct mechanisms of action, including TNF inhibitors, anti-IL 17 agents, anti-IL 23 agents, and JAK inhibitors. “Very D2T PsA” referred to those who experienced failure with two or more b/tsDMARDs within less than two years of follow-up. Their findings revealed that in the “D2T” group, approximately 40.4% of patients underwent at least one therapeutic switch due to inadequate control of psoriasis, in contrast to 18.8% among the non-'D2T' group (p=0.005). Multivariate analysis revealed that the “D2T” population was 2.75 times more likely to discontinue a bDMARD due to poor psoriasis control, with a 95% confidence interval of 1.23 to 6.14 (p=0.013). Additionally, “D2T” PsA was linked to a greater incidence of axial involvement and structural damage. Patients classified as “Very D2T” PsA were more likely to present a higher BMI and exhibit axial involvement.^24,30^

Interestingly, a recent study from the Greek multicentre PsA registry also identified poor dermatological response and axial disease as independent risk factors for D2T PsA. Consistent with the findings of the French study, obesity was also associated with D2T PsA.^29^

In the study focusing on Axial Spondyloarthritis, “D2T” axSpA was defined as the failure to respond to at least two b/tsDMARDs with differing mechanisms of action (MoA), including TNF inhibitors, IL-17 inhibitors, and JAK inhibitors. Very D2T axSpA was defined as the failure to respond to at least two b/tsDMARDs within a period of less than two years.

Notably, the “D2T” AxSpA group exhibited a significantly longer duration of disease in comparison to the non-D2T group (p=0.006). Peripheral involvement was notably higher in the “D2T” group, affecting 34.9% of patients compared to 21.4% in the non-“D2T” group (p=0.015). Moreover, patients in the “D2T” group presented higher baseline BASDAI levels (mean BASDAI 63.7 versus 58.8, p=0.015). Fibromyalgia was observed more frequently in the “D2T” group compared to the non-D2T group (p < 0.001). Approximately one out seven (14%) “D2T” patients had a comorbidity such as multiple sclerosis, Inflammatory bowel disease (IBD), recurrent uveitis or solid cancer within the past five years, which serves as a contraindication for the use of anti-TNF or anti-IL-17 inhibitors.

On the other hand, patients in the “Very D2T” AxSpA group exhibited a notably higher prevalence of Inflammatory bowel disease (IBD) at baseline —41.7% compared to 3.1% in the non-D2T group (p<0.001)— and, to a lesser extent, uveitis—45.5% versus 11% (p=0.020). Interestingly, none of the “Very D2T” patients had a diagnosis of fibromyalgia.^25^

These findings underscore the diverse clinical characteristics and challenges faced by patients with PsA and axSpA who require multiple treatment switches, highlighting the importance of tailored therapeutic approaches in managing these complex conditions. Building upon previous research, mainly in the field of inflammatory bowel disease (IBD), a team of researchers from Brazil, led by Pimentel et al., sought to evaluate Anti-Infliximab Antibodies (ADA) in patients with AS. Their study aimed to explore the potential role of ADA as a biomarker, extending beyond its well-established impact on drug survival. Specifically, they investigated its influence on treatment failure, infusion reactions, tapering strategies, and the efficacy of second TNF inhibitor therapy in AS patients.^26^

Infusion reactions were notably more frequent among patients positive for anti-Infliximab antibodies (anti-IFX) at both 22–24 weeks (p=0.020) and 48–54 weeks (p=0.034). Furthermore, the presence of anti-IFX antibodies between 48–54 weeks correlated with treatment failure (46.7% compared to 13.8%; p=0.028) and significantly reduced the overall survival rates of Infliximab (54.9 months [95% CI 26.3–83.4] vs. 148.9 months [95% CI 123.5–174.4]; p<0.001).

Interestingly, among patients in the tapering group (n=24), the presence of anti-IFX antibodies was linked to a shorter survival of this strategy, lasting only 9.9 months [95% CI 4.0–15.8] compared to 63.4 months [95% CI 27.9–98.8] in those without antibodies (p=0.004). In contrast, among patients who experienced treatment failure with Infliximab (n=29), those who tested positive for anti-IFX at the time of switching to a second TNF inhibitor demonstrated a significantly better clinical response at both 3 months (83.3% vs. 27.3%; p=0.005) and 6 months (83.3% vs. 36.4%; p=0.017).^26^ These findings underline the multifaceted impact of anti-IFX antibodies on treatment outcomes and highlight their potential utility as a biomarker in guiding therapeutic decisions for AS patients.

JAK inhibitors (JAKi) have emerged as a promising treatment option for a range of inflammatory conditions, including PsA. While the efficacy and safety of JAK inhibitors in rheumatoid arthritis are increasingly understood, their use in PsA is still a developing field. The “Jak-spot” study, an international collaboration of registers, presented data at ACR 2023, providing valuable insights into the utilisation of JAK inhibitors in patients with PsA.^27^

In the analysis encompassing 11,939 treatment courses from 12 registers where JAKi were prescribed for PsA, a diverse cohort was examined. Among these treatment courses, 582 were initiated with JAKi, predominantly tofacitinib (67%) and upadacitinib (27%), with baricitinib accounting for a smaller proportion (6%). Patients starting JAKi tended to exhibit more challenging disease characteristics, including longer disease duration (over 9 years), older age, a higher frequency of prior biologic DMARD (bDMARD) use (52% with 3 or more previous bDMARDs), and increased concomitant glucocorticoid therapy. Despite variations in the use of specific JAKi agents across countries, patient characteristics remained consistent.

The one-year crude drug retention rate for JAKi was 65%, a significantly lower rate compared to other modes of action biologic DMARDs (OMA) at 74% and TNF inhibitors at 77%. However, adjusted analyses demonstrated that JAK inhibitors showed comparable drug maintenance rates to TNF inhibitors (TNFi) and other targeted biologic therapies such as IL-17 and IL-12/23 inhibitors. Conversely, patients initiating OMA, such as apremilast, experienced significantly shorter periods of drug maintenance. In particular, the estimated hazard ratios for treatment discontinuation compared to TNF inhibitors were as follows: IL-17 inhibitors had an HR of 0.93 (0.77 – 1.12), JAK inhibitors had an HR of 1.19 (0.92 – 1.54), IL-12/23 inhibitors had an HR of 1.22 (0.97 – 1.54), and other modes of action biologic DMARDs had an HR of 1.54 (1.25 – 1.91).^27^

Exciting insights emerged from this year's ACR convergence regarding Izokibep, a novel IL-17A inhibitor. Unique for its high IL-17A binding affinity, small molecular size at 18.6 kD, and an albumin binding site facilitating access to inflamed tissues, Izokibep presents a promising approach in the treatment landscape. The presentation showcased results from a phase 2 trial in PsA, shedding light on its efficacy across arthritis and skin composite endpoints, alongside longer-term safety considerations. In the multicentre trial (NCT04713072) evaluating Izokibep, doses of 80 mg every 2 weeks (Q2W) or 40 mg Q2W were administered up to 46 weeks or study termination.

Eligible patients satisfied the CASPAR criteria, exhibiting at least three swollen and three tender joints, and had previously failed to respond adequately to NSAIDs, csDMARDs, or TNF inhibitors. Randomising 135 patients in a 1:1:1 ratio, 44 received 40 mg Q2W, 47 received 80 mg Q2W, and 44 initially received placebo before switching to 80 mg Q2W at week 16.

Beyond week 16, patients receiving the 80 mg dosage continued to show improvement, whereas those in the 40 mg group maintained a largely stable condition. Remarkably, a substantial number of patients in both the 80 mg group and the placebo to 80 mg switchers group achieved notable outcomes through week 46. Specifically, 65% of patients in the 80 mg group achieved DAPSA low disease activity or remission status, as did 71% of those who switched from placebo to 80 mg. Furthermore, 57% of patients in the 80 mg group and 67% of those in the placebo to 80 mg switchers group achieved minimal disease activity (MDA) thresholds. Additionally, 52% of patients in the Izokibep 80mg group achieved ACR70 response, while an impressive 71% of patients attained PASI100 response.

Furthermore, the study demonstrated sustained high rates of enthesitis resolution with Izokibep treatment. Initially, 80 mg dosage showed promising results with 89% of patients experiencing resolution of enthesitis according to the LEI scale by week 16. Impressively, this resolution was maintained throughout the study period. Additionally, SPARCC enthesitis resolution rates improved over time, with 72% of patients achieving resolution by week 46.^28^

This data heralds a potentially impactful addition of Izokibep to the therapeutic armamentarium for PsA, warranting further exploration and anticipation in the rheumatology community.

## CONCLUSION

Both EULAR and ACR 2023 have provided valuable insights and advancements in the understanding and treatment of Spondyloarthritis. The studies presented highlight the dynamic nature of these conditions and the ongoing effort to enhance diagnostic precision and therapeutic effectiveness. From groundbreaking basic science research elucidating the complex pathophysiology of axSpA to clinical trials showcasing novel treatments such as JAK inhibitors and the promising IL-17A inhibitor Izokibep, the field is witnessing significant progress. Biologic DMARDs continue to be pivotal in alleviating pain, fatigue, and enhancing quality of life in axSpA patients. Importantly, long-term studies suggest that while these therapies are generally safe, continuous monitoring for adverse events remains crucial.

The challenges of “difficult-to-treat PsA” and the importance of early diagnosis and timely treatment initiation have been spotlighted, suggesting a pivotal shift towards a more aggressive and tailored management approach. Additionally, the evolving understanding of treatment responses across different baseline characteristics and the impact of concomitant conditions like IBS and valvular heart disease provide a deeper layer of complexity in treatment planning. This aligns with the broader therapeutic strategies across rheumatic diseases aiming to optimise long-term health and quality of life for patients. The future of managing Spondyloarthritis appears promising, with the potential to significantly alter the disease trajectory and enhance the quality of life for those impacted.
